# Better Adherence to the Mediterranean Diet Could Mitigate the Adverse Consequences of Obesity on Cardiovascular Disease: The SUN Prospective Cohort

**DOI:** 10.3390/nu7115457

**Published:** 2015-11-05

**Authors:** Sonia Eguaras, Estefanía Toledo, Aitor Hernández-Hernández, Sebastián Cervantes, Miguel A. Martínez-González

**Affiliations:** 1 Servicio Navarro de Salud-Osasunbidea-IdiSNA, Navarra Institute for Health Research, 31002 Pamplona, Spain; seguaras@alumni.unav.es; 2 Department of Preventive Medicine and Public Health, Navarra Institute for Health Research, University of Navarra-IdiSNA, 31008 Pamplona, Spain; etoledo@unav.es (E.T.); aitorhernandez86@gmail.com (A.H.-H); sebcervantes@gmail.com (S.C.); 3 Biomedical Research Center Network on Obesity and Nutrition (CIBERobn) Physiopathology of Obesity and Nutrition, Instituto de Salud Carlos III, 28029 Madrid, Spain; 4 Department of Cardiology, Navarra Institute for Health Research, University Clinic of Navarra-IdiSNA, 31008 Pamplona, Spain; 5 Department of Radiology, Hospital of Navarra, Servicio Navarro de Salud-Osasunbidea-IdiSNA, Navarra Institute for Health Research, 31008 Pamplona, Spain

**Keywords:** obesity, Mediterranean diet, prospective cohort study, cardiovascular disease, SUN project

## Abstract

Strong observational evidence supports the association between obesity and cardiovascular events. In elderly high-risk subjects, the Mediterranean diet (MedDiet) was reported to counteract the adverse cardiovascular effects of adiposity. Whether this same attenuation is also present in younger subjects is not known. We prospectively examined the association between obesity and cardiovascular clinical events (myocardial infarction, stroke or cardiovascular death) after 10.9 years follow-up in 19,065 middle-aged men and women (average age 38 year) according to their adherence to the MedDiet (<6 points or ≥6 points in the Trichopoulou’s Mediterranean Diet Score). We observed 152 incident cases of cardiovascular disease (CVD). An increased risk of CVD across categories of body mass index (BMI) was apparent if adherence to the MedDiet was low, with multivariable-adjusted hazard ratios (HRs): 1.44 (95% confidence interval: 0.93–2.25) for ≥25 – <30 kg/m^2^ of BMI and 2.00 (1.04–3.83) for ≥30 kg/m^2^ of BMI, compared to a BMI < 25 kg/m^2^. In contrast, these estimates were 0.77 (0.35–1.67) and 1.15 (0.39–3.43) with good adherence to MedDiet. Better adherence to the MedDiet was associated with reduced CVD events (*p* for trend = 0.029). Our results suggest that the MedDiet could mitigate the harmful cardiovascular effect of overweight/obesity.

## 1. Introduction

The prevalence of obesity is increasing globally, and it is one of the major public health problems in most countries. According to WHO, in the last three decades the prevalence of obesity has doubled worldwide. As a result, the majority of world population currently live in countries where overweight and obesity cause more deaths than insufficient weight [1].

Excess body weight is likely to be associated with clinical cardiovascular disease (CVD) even at moderate levels of overweight and independently of traditional cardiovascular risk factors [2,3,4,5]. On the other hand, dietary habits play an important role as determinants of optimal health and—more importantly—they may be especially useful for the prevention of CVD [6,7]. There is compelling evidence that the traditional Mediterranean diet (MedDiet) has beneficial effects against all-cause mortality and clinical cardiovascular events [8,9,10]. In the context of the current pandemic of overweight/obesity, it is likely that dietary habits in line with the traditional Mediterranean dietary pattern may attenuate the well-known detrimental effects of adiposity on cardiovascular risk. In this line of thought, a recent study conducted in elderly high-risk subjects suggested that the MedDiet could counteract the adverse cardiovascular effects of an increased body weight [11]. It is not known whether this attenuation by the MedDiet of the harmful cardiovascular effects of even a moderate degree of excess adiposity is also present in younger subjects at lower cardiovascular risk. We prospectively assessed the association between obesity and incidence of major clinical cardiovascular events within categories of adherence to the MedDiet in a sample of 19,065 highly educated men and women followed-up for a mean period of 10.9 years, with mean age of 38 years old and a low predicted risk of cardiovascular disease at baseline. 

## 2. Experimental Section

### 2.1. Study Population

The SUN project is a multipurpose prospective Spanish cohort study entirely composed of university graduates. This cohort was designed to assess associations of diet or lifestyle with the incidence of several chronic diseases and mortality. The study protocol was approved by the Institutional Review Board of the University of Navarra. The design, methods and objectives of the SUN project have been described previously [12].

The recruitment of participants started in December 1999. It is a dynamic cohort permanently open to recruitment of new participants. Up to December 2014, 22,175 participants had answered the baseline questionnaire. Follow-up information is collected through self-administered questionnaires sent biennially by mail.

**Figure 1:**
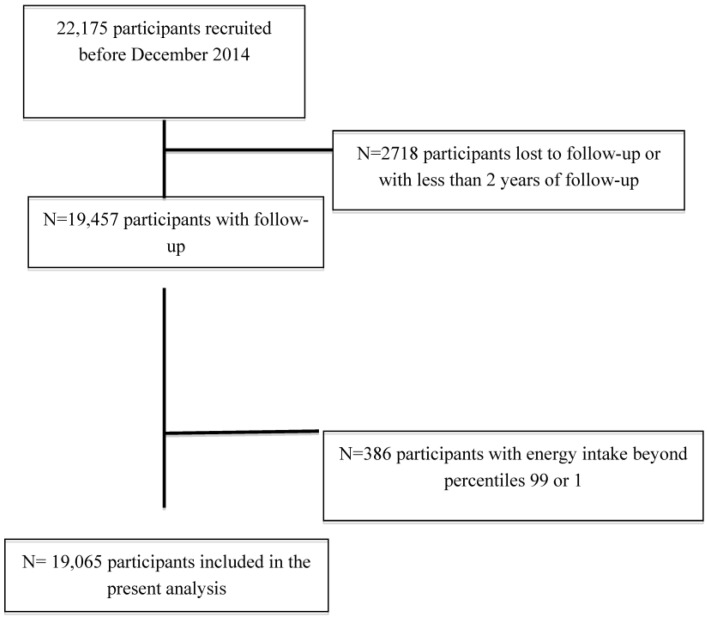
Flow-chart of participants in the SUN Project, 1999–2014.

For the present analysis, 2718 participants were lost to follow up or were recruited for the cohort only for a short period (less than 2 years) and were therefore excluded from our analyses; in addition, 386 participants with total daily energy intake beyond percentiles 1 or 99 were also excluded. Thus, our final sample included 19,065 participants (Figure 1).

### 2.2. Anthropometric Variables

Participants’ weight was recorded at baseline. Reliability and validity of self-reported weight has been previously assessed in a subsample of the cohort, and has shown a high correlation with directly measured weight (*r* = 0.99; 95% CI: 0.99, 0.99) and a mean relative error of 1.45% [13]. Body mass index (BMI), defined as weight in kilograms divided by the square of height in meters, was computed in the baseline questionnaire. Reliability of self-reported weight and height used to calculate BMI has been previously assessed [13] (*r* = 0.94; 95% CI: 0.91, 0.97). Mean relative error in self-reported BMI was 2.64%.

### 2.3. Dietary Assessment

Usual diet was assessed at baseline with a validated semi-quantitative 136-item food-frequency questionnaire (FFQ) [14,15]. Each item included a typical portion size, and consumption frequencies were grouped in nine categories that ranged from “never or almost never” to “≥6 times/day.” A trained team of dietitians updated the nutrient data bank using the latest available information included in food composition tables for Spain [16,17]. We used the Mediterranean Diet Score (ranging from 0 to 9 points) proposed by Trichopoulou *et al.* [18] to classify participants according to their baseline adherence to the Mediterranean diet [19]. One point was assigned to persons whose consumption was at or above the sex-specific median of components frequently consumed in the context of traditional Mediterranean diet (vegetables, fruits/nuts, legumes, fish/seafood, cereals, and monounsaturated/saturated (MUFA/SFA) fat ratio). The participant received also 1 point if her or his intake was below the median for the 2 components less frequently consumed in the context of traditional Mediterranean diet (meat and dairy products). For ethanol, 1 point was assigned only for moderate amounts of intake (5–25 g/day for women or 10–50 g/day for men.). We dichotomized adherence to MedDiet into 2 categories (<6 and ≥6 points in the Trichopoulou’s score).

### 2.4. Other Covariates

We used standardized questionnaires included in the baseline questionnaire to gather information about socio-demographic parameters (sex, age), anthropometric measurements (weight, BMI) and health-related habits (smoking status, physical activity, sedentary lifestyle). Information on physical activity was collected at baseline through a previously validated questionnaire that contained time spent in 17 different activities. Physical activity was expressed in metabolic equivalent tasks (METs = time spent at each activity in hours/week multiplied by its typical energy expenditure) [20].

### 2.5. Outcome Assessment

Incidence of cardiovascular events, defined as non-fatal myocardial infarction, non-fatal stroke reported by participants on a follow-up questionnaire or deaths due to cardiovascular disease, was the primary endpoint. An expert panel of physicians, blinded to information on diet, anthropometric indexes and risk factors, reviewed medical records of participants and adjudicated events applying universal criteria for myocardial infarction and clinical criteria for the other outcomes. A non-fatal stroke was defined as a focal neurological deficit of sudden onset and vascular mechanism lasting >24 h. Cases of fatal stroke were documented if there was evidence of a cerebrovascular mechanism. Deaths were reported to our research team by the participants’ next of kin, work associates and postal authorities. For participants lost to follow-up, we consulted the National Death Index every 6 months to identify deceased cohort members and to obtain their cause of death. Cases of fatal CHD or stroke reported by families or postal authorities were confirmed by a review of medical records with permission of the next of kin.

### 2.6. Statistical Analyses

Analyses were performed with STATA version 12.0 (StataCorp, College Station, TX 77840, USA). For descriptive purposes, we calculated means, standard deviations, proportions, medians, and percentiles of baseline characteristics across levels of adherence to the Mediterranean diet. We used Cox regression models to assess hazard ratios (HRs) and their 95% confidence intervals (CIs) for total CVD events across categories of BMI (cut off points: 25 and 30 kg/m^2^). We used age as the underlying time variable and stratified all analyses for age groups. To assess attenuation by the MedDiet of the harmful effect of obesity, we stratified results for baseline adherence to the MedDiet (categorized into two groups: low adherence (<6 points) and high adherence (≥6 points)). We included as covariates potential confounders such as age (underlying time variable, plus stratification), sex, smoking status (3 categories), physical activity during leisure time, baseline hypertension status, baseline hypercholesterolemia status, diabetes, years of university education, and previous history of CVD (present in only 173 participants) in multivariable analyses.

We also assessed incidence of cardiovascular events according to four categories of adherence to MedDiet: low (2 points), low-moderate (3–4 points), moderate-high (5–6 points) and high (7–9 points). In addition, linear trend test were also conducted using Trichopoulou’s score of adherence to MedDiet as a continuous variable.

## 3. Results

Table 1 shows the baseline characteristics of participants according to their baseline adherence to MedDiet and stratified by their baseline BMI. In addition to having a high level of education, SUN cohort participants were relatively young at baseline (mean age 38.4 years), more likely to be women (60.5%) and non-obese (average BMI = 23.5 kg/m^2^; 95.3% of participants had a BMI < 30 kg/m^2^). However, participants with better adherence to MedDiet were older, more physically active, more likely to be men and married and to follow special diets, and less likely to snacks between meals. They were also less likely to be current smokers but more prone to being former smokers. In addition, those participants with initial good adherence to the MedDiet were more likely to have a previous diagnosis of hypercholesterolemia, hypertriglyceridemia, diabetes or hypertension.

**Table 1:** Baseline characteristics of participants according to Mediterranean diet adherence and BMI.

	Low Adherence to MedDiet (<6/9)			High Adherence to MedDiet (≥6/9)		
	BMI <25	BMI 25–30	BMI >30	BMI <25	BMI 25–30	BMI >30
N	10,169	3396	663	3208	1396	233
Age (years)	35 (10)	43 (13)	45 (12)	39 (12)	48 (12)	48 (13)
Women (%)	74.3	30.4	32.0	71.7	28.2	23.6
BMI (kg/m^2^)	21.6 (1.9)	26.9 (1.3)	32.5 (2.4)	21.9 (1.9)	26.9 (1.3)	32.7 (3.0)
Previous history of CVD (%)	0.4	1.3	1.5	0.8	3.0	4.3
Energy intake (kcal/day)	2479 (766)	2353 (756)	2386 (812)	2698 (786)	2633 (766)	2715 (800)
Leisure-time physical activity	20.6 (22.1)	20.4 (21.7)	15.2 (17.7)	26.8 (26.0)	24.6 (23.3)	19.7 (18.0)
Marital status						
Single	53.5	30.9	27.8	43.6	19.9	5.7
Married	42.4	63.9	65.6	50.7	74.4	70.4
Others	4.0	5.2	6.6	5.7	5.8	7.7
Smoking current smokers (%)	22.9	20.4	19.6	20.8	17.9	18.0
Former smokers (%)	22.6	34.9	39.5	30.5	44.4	48.1
Baseline hypercholesterolemia (%)	11.3	22.5	32.3	17.8	33.0	38.2
Baseline triglycerides (%)	2.9	12.1	23.5	4.4	16.3	30.9
Diabetes at baseline (%)	0.9	2.8	5.7	1.6	3.5	7.3
Hypertension at baseline (%)	3.0	12.5	25.9	5.2	19.3	32.6
Years of university education	5.0 (1.47)	5.2 (1.60)	5.0 (1.51)	5.0 (1.50)	5.3 (1.72)	5.1 (1.50)
Leisure-time spent sitting down, h/week.	3.8 (1.72)	3.8 (1.91)	3.8 (2.06)	3.9 (1.86)	4.0 (1.83)	4.1 (2.01)
TV watching, h/week.	1.6 (1.23)	1.7 (1.14)	1.8 (1.18)	1.6 (1.16)	1.7 (1.09)	1.8 (1.17)
Between-meal snacking (%)	35.2	34.7	51.3	27.9	29.3	40.8
Following special diets (%)	5.6	9.6	15.1	9.2	12.8	19.3

BMI: body mass index; SD: standard deviation; CVD: cardiovascular disease (acute coronary syndromes or stroke).

We observed 152 incident cases of CVD (56 non-fatal myocardial infarctions, 30 non-fatal strokes and 66 cardiovascular deaths) after a mean of 10.9 years of follow–up. As shown in Table 2, we assessed the relationship between classical categories of BMI and risk of CVD clinical events according to categories of baseline adherence to MedDiet (<6 and ≥6 points in the Trichopoulou’s score). An increased risk of CVD events across categories of BMI was apparent in the low adherence to MedDiet group. Within each group of BMI, the group with lesser conformity to MedDiet had higher rates of age-adjusted CVD than the group with good adherence to MedDiet.

**Table 2:** Relative risk (hazard ratios and 95% confidence intervals) of incident cardiovascular disease (myocardial infarction, stroke or cardiovascular death) according to baseline body mass index and adherence to MedDiet. The SUN project 1999–2014.

	Low Adherence to MedDiet (<6/9)			High Adherence to MedDiet (≥6/9)		
Body mass index	<25	25–30	>30	<25	25–30	>30
*n*	10,169	3396	663	3208	1396	233
Median body mass index	21.6	26.9	32.5	21.9	26.9	32.7
events	38	59	15	15	19	6
Person-years	95,620	30,961	5814	28,260	11,917	1789
Age-adjusted rate/10^5^ (95% CI)	40 (28–55)	74 (49–113)	97 (53–177)	31 (17–57)	43 (25–76)	86 (36–206)
Sex-, age-adjusted HR	1 (ref.)	1.52 (0.99–2.35)	2.14 (1.14–4.00)	1 (ref.)	0.97 (0.48–1.95)	1.77 (0.61–5.17)
Multivariable-adjusted HR *	1 (ref.)	1.44 (0.93–2.25)	2.00 (1.04–3.83)	1 (ref.)	0.77 (0.35–1.67)	1.15 (0.39–3.43)

* Adjusted for age (underline time variable plus stratification), sex, smoking, baseline hypercholesterolemia, hypertension, leisure-time physical activity, hypertension, diabetes and previous history of cardiovascular disease. Robust standard errors were used. *p* for interaction (BMI × MedDiet) = 0.10.

Among participants with low adherence to the MedDiet and compared to participants with a BMI < 25 kg/m^2^, multivariable-adjusted hazard ratios (95% confidence intervals) of CVD were 1.44 (0.93–2.25) for participants with a BMI ≥25 – <30 kg/m^2^, and 2.00 (1.04–3.83) for participants with BMI > 30 kg/m^2^. On the other hand, in the group with good adherence to the MedDiet, multivariable-adjusted hazard ratios (HRs) were 0.77 (0.35–1.67) and 1.15 (0.39–3.43), respectively. Therefore, there was a trend towards an attenuation of the harmful effects of obesity by the MedDiet. However, the *p* value for the multiplicative interaction (to test for an effect beyond the multiplication of both independent effects) was not statistically significant (*p* = 0.10).

We also confirmed an inverse association between adherence to MedDiet and CVD events (Table 3), which was previously reported when the accrual in this cohort, measured as person-years, was smaller [21]. In this updated analysis, participants with the highest adherence (score >7) showed a 53% lower risk of cardiovascular events as compared to participants in the lowest adherence category (score <2), after adjustment for potential confounders. The inverse linear trend for the association between adherence to the MedDiet and CVD was statistically significant (*p* = 0.029). A two-point increment in the Mediterranean-diet score was associated with a 7% relative reduction in CVD risk.

**Table 3:** Relative risk (Hazard Ratios and 95% confidence intervals) of incident cardiovascular disease (myocardial infarction, stroke or cardiovascular death) according to baseline adherence to the MedDiet. The SUN project 1999–2014.

Adherence to the Mediterranean Diet	Low 0–2	Low-Moderate 3–4	Moderate-High 5–6	High 7–9	*p* for Trend	For Each +2 Points
*n*	3334	7160	6431	2140		
Incident cases of CVD	23	57	51	21		
Person-years	32,001	66,900	57,120	18,341		
Sex-, age-adjusted HR	1 (ref.)	0.88 (0.53–1.46)	0.68 (0.40–1.15)	0.58 (0.31–1.09)	0.097	0.95 (0.91–0.99)
Multivariable-adjusted HR *	1 (ref.)	0.81 (0.48–1.38)	0.58 (0.34–1.00)	0.47 (0.25–0.89)	0.029	0.93(0.89–0.98)

* Adjusted for age (underline time variable plus stratification), sex, smoking, baseline hypercholesterolemia, hypertension, leisure-time physical activity, hypertension, diabetes and previous history of cardiovascular disease. Robust standard errors were used. *p* for interaction (BMI × MedDiet) = 0.10.

**Figure 2:**
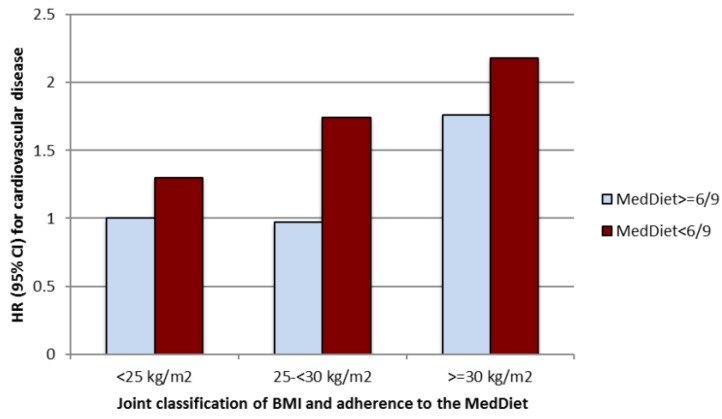
Relative risk of cardiovascular disease (HR and 95% confidence intervals) in the SUN project according to baseline body mass index and adherence to MedDiet.

Figure 2 presents multivariable-adjusted hazard ratios for the joint classification according to both values of BMI (3 groups with the following 2 cut-off points: 25 and 30 kg/m^2^) and adherence to MedDiet (two categories: low adherence (<6 points) and high adherence (≥6 points)). The reference category was the group with low BMI (<25) and high adherence to MedDiet (score ≥6). In the group with better adherence to MedDiet, the risk of CVD was lower than in the poor adherence group across all BMI categories.

## 4. Discussion

This study contributes to support the evidence that closer adherence to the MedDiet could counteract some of the adverse cardiovascular effects of overweight/obesity, not only in elderly persons at high cardiovascular risk but also in young, healthier and highly-educated persons. Though we did not find a statistically significant interaction and the *p* value for the product-term only approached statistical significance, a biological interaction was suggested by our results. In fact, the effect of increased adiposity was mitigated in subjects with high adherence to MedDiet.

There is evidence that excess body weight is associated with an increased risk of CVD [22,23]. Excess weight is associated with both subclinical metabolic and vascular dysfunction that, with the passage of time, lead to an increased risk of CV events, due to a state of low-grade inflammation that increases cardiovascular risk [24,25,26]. Therefore, it seems biologically plausible that the MedDiet’s anti-inflammatory effects [27] could counter-balance the detrimental effects of obesity-associated low-grade inflammation.

The association between overweight and CVD is not as universally acknowledged as that between obesity and CVD. For instance, a recently published meta-analysis suggested that overweight subjects (defined as those with a BMI of 25 to 30 kg/m^2^) have lower all-cause mortality as compared to normal weight subjects. In addition, persons with grade I obesity (BMI between 30 and 35 kg/m^2^) had a significantly lower risk of mortality [28]. In contrast, in our study an increased risk of CVD was observed both in obese and overweight subjects. A possible explanation for this discrepancy lies in the presence of potential biases that may have attenuated the association between obesity and CVD risk in previous studies conducted with older participants, such as higher rate of tobacco use and presence of preclinical diseases. We were able to avoid this problem as our examined sample was composed of healthy and young individuals with a low prevalence of pre-existing disease.

We used BMI to measure excess of body weight, as it is the most widely used and accepted index for assessing obesity. However, anthropometric indexes such as the waist-to-height (WHtR) or waist circumference (WC) have been shown to have advantages as predictors of CVD [29,30]. Nevertheless, weight and height are objective and reproducible measures that have been accurately reported by our participants in a validation study of self-reported measurements [13].

Previous studies have suggested the beneficial metabolic effects of the MedDiet, regardless of abdominal adiposity [31,32,33,34]. Our results support a previous study that suggested a beneficial effect from the MedDiet in obese subjects [11]. In addition, we have found evidence that extends this benefit to overweight subjects.

There are several strengths in our research that deserve to be mentioned. We used a large sample of participants with a high retention rate. The prospective nature of our study with its long follow-up period allowed us to detect CVD events and avoid reverse causation bias in the reported associations. In addition, multiple-adjusted models enabled us to control for a wide array of potential confounders.

We find that our study has a strong internal validity thanks to a high retention rate and reliable self-reported measures reported by highly educated participants. In addition, internal validity is reinforced by restriction to subjects with high educational levels so that the risk for confounding by education or socio-economic levels is minimized. On the other hand, we acknowledge some limitations of our study. First, the information on several variables was collected through self-reporting. However, parameters such as self-reported usual diet, weight or BMI have been previously validated [13], therefore decreasing risk of residual misclassification. In addition, outcomes were confirmed by a panel of physicians after blindly reviewing participants’ medical records. Second, the product-term in the fully adjusted model showed a non-significant interaction. The relatively small number of events observed in our cohort could explain the lack of statistical significance of this interaction product-term. However, this low number of cardiovascular events should not be surprising given the young age and healthy characteristics of our participants.

## 5. Conclusions

Our study suggests benefits from the MedDiet as an effective tool for counteracting the detrimental effects of obesity on cardiovascular health.
